# Emerging trends in enamel remineralization

**DOI:** 10.6026/973206300220373

**Published:** 2026-01-31

**Authors:** Mehar Kaur, Barbara Vigas, Syeda Jaffari, Salina Manandhar, Tuga Elhaboub, Oreoluwa Tedela

**Affiliations:** 1 Department of Dental Surgery, Sudha Rustagi College of Dental Sciences & Research, Faridabad, India; 2 Department of Dental Prosthetics, Faculdade do Amazonas (IAES), Manaus, AM, Brazil; 3 Department of Dental Surgery, Islamabad Medical and Dental College, Islamabad, Pakistan, India; 4 Department of Dental Surgery, Rangpur Community Medical and Dental College and Hospital, Rangpur, Bangladesh; 5 Department of Dental Surgery, Alneelain University, Khartoum, Sudan; 6 Department of Dental Surgery, Dalhousie University, Halifax, Nova Scotia, Canada

**Keywords:** Casein phosphopeptide-amorphous calcium phosphate (ACP-CPP), biomimetic agents, amelogenin-derived peptides, chitosan hydrogels, PAMAM dendrimers, nano-technology

## Abstract

The enamel of our teeth is heavily mineralized and undergoes repeated cycles of demineralization and remineralization. Despite long- term use of fluorides, ACP-CPP and bioactive glass as remineralizing agents, their limited properties prevent complete restoration of the enamel's hierarchical structure, leading to the introduction of biomimetic agents that mimic the natural formation of enamel while also exhibiting antibacterial effects. Biomimetic strategies include amelogenin-derived peptides, chitosan hydrogels and Poly (amidoamine) dendrimers (PAMAM). Nanotechnology integrates nano-hydroxyapatite and calcium salts into weakened enamel. At the same time, smart materials incorporate pH-responsive composites and ion-releasing sealants that target remineralization and exhibit antimicrobial effects under cariogenic conditions. While these approaches show promising results, their routine clinical adoption requires additional cost, scalability and regulatory challenges.

## Mechanisms of enamel demineralization and remineralization:

Tooth enamel is composed primarily of hydroxyapatite (HA) crystals and is highly mineralized. Despite its strength, enamel undergoes demineralization when exposed to acids from bacterial metabolism or dietary sources, dissolving HA and weakening the structure [1]. Remineralization is a natural repair process that restores lost minerals. Saliva plays a central role by buffering pH and delivering calcium and phosphate. Fluoride enhances this by forming fluorapatite, a more acid-resistant mineral [2]. Recent studies highlight biomimetic alternatives to fluoride. For example, nano- hydroxyapatite (nHA) toothpaste can remineralize early enamel lesions by penetrating surface porosities [3]. Zinc-carbonate hydroxyapatite also demonstrates effectiveness in remineralizing enamel and reducing hypersensitivity, offering a fluoride-free option [4]. Chitosan, a naturally derived biopolymer, supports enamel repair by stabilizing mineral ions and serving as a scaffold for new mineral deposition. In dentin, carboxymethyl chitosan has aided collagen mineralization [5]. Peptide-based therapies, especially those using amelogenin-derived peptides, mimic the natural enamel formation process. For example, the recombinant amelogenin peptide TRAP has shown the ability to promote remineralization of early enamel lesions in situ, especially when integrated with delivery systems such as hydrogels [6]. Maintaining enamel integrity depends on balancing mineral loss and gain. Preventive care, fluoride exposure, diet control and new biomimetic agents play a pivotal role in managing caries risk.

## Conventional remineralization agents:

Fluoride has been a powerful remineralizing agent for many years. Fluorides work mainly by making fluorapatite crystals more acid- resistant than hydroxyapatite alone. Fluorides help remineralization by attracting more calcium and phosphate together. It diminishes the production of pyruvate in the glycolytic pathway of bacteria. Additionally, it holds on to the tooth surface strongly, hampering the demineralization of tooth structure [7]. Fluoride toothpastes in conjunction with fluoride mouth rinses can significantly improve the remineralization of incipient caries. The frequency of using a mouth rinse is the key determinant in remineralization [8]. Calcium salts, for example, hydroxyapatite and calcium carbonate, are used to remineralize early enamel lesions. According to M Carey, scanning electron microscopy showed smoother and stronger enamel. The enamel appeared to be more intact, revealing deposition of calcium salts by chemically replenishing lost salts. Calcium salts also physically plug in and minimize microscopic defects, thus forming new mineral bridges [9]. In hydroxyapatite, calcium phosphate forms its main constituent. Plaque and salivary levels of calcium and phosphate determine the extent of demineralization and remineralization of enamel. For remineralization, an ideal ratio of calcium to phosphate is 1.6. The naturally existing ratio for calcium to phosphate is 0.3; therefore, supplementing additional concentration of calcium can boost remineralization. Beta tricalcium phosphate is another common compound used for remineralization. It is used in combination with fluoride. In toothpaste, a protective barrier envelopes this compound to minimize any chance of premature reaction with fluoride. During brushing, this barrier is removed while supplying saliva with calcium, phosphate and fluoride ions. Beta TCP is functionalized with either organic or inorganic constituents to form functionalized TCP. Functionalized TCP is a more stable form of TCP and is engineered at low doses [10]. ACP-CPP has been used widely as a non-fluoride alternative to promote remineralization of enamel. It was discovered by Prof. Reynold at the School of Dental Science in Australia. CPP is used to stabilize the ACP because of its ability to lose calcium and phosphate during precipitation. CPP increases the bioavailability of calcium and phosphate ions from milk and other dairy products. Additionally, CPP also increases the efficacy of fluoride by constantly maintaining fluoride concentration in solution. Laser autofluorescence showed that using ACP- CPP with fluoride together has a synergistic effect. Calcium phosphate is generally insoluble and tends to form a crystalline structure at neutral pH. CPP helps to keep calcium phosphate in a soluble amorphous state, which is then easily taken up by tooth enamel and aids in remineralization and decreases demineralization. Primarily, ACP is derived from two salts, calcium sulphate and potassium phosphate, which were created by Dr. Ming S. Tung. Amorphous calcium phosphate has proven to be a powerful remineralizing agent when used in conjunction with CPP [11]. Furthermore, bioactive glass was created by Dr. Larry Hench in the 1960s. It acts as a biomimetic mineralizing agent. It takes around 12-24 hours to fully take place in a series of chemical reactions to form a 100-150 micrometer layer of carbonated hydroxyapatite. Building upon these conventional strategies, biomimetic approaches aim not only to replace lost minerals but also to recreate the natural microstructure of enamel [12].

## Biomimetic approaches in enamel remineralization:

Biomimetic approaches in enamel remineralization aim to replicate the natural biomineralization process, restoring enamel's hierarchical structure and mechanical properties. Unlike conventional methods like fluoride, which primarily enhance mineral deposition, biomimetic strategies mimic the organic matrix-mediated crystal growth seen during enamel development, offering innovative solutions for caries prevention and repair [13].

## Amelogenin-derived peptides and chitosan hydrogels:

Amelogenin-derived peptides, such as P26 and P32, play a critical role in guiding hydroxyapatite (HA) crystal formation. Delivered via chitosan hydrogels, these peptides promote organized crystal growth, enhancing enamel hardness and occluding dentin tubules *in vitro* [14]. Chitosan, a biocompatible polysaccharide derived from chitin, serves as a scaffold, stabilizing calcium and phosphate ions to facilitate remineralization while providing antimicrobial properties to reduce bacterial adhesion [5]. Studies show that carboxymethyl chitosan combined with amorphous calcium phosphate (ACP) forms ordered apatite crystals on demineralized enamel surfaces, closely mimicking natural enamel's microstructure. This approach has demonstrated improved surface microhardness in laboratory models, suggesting potential for clinical applications [15].

## Self-assembling peptides:

Self-assembling peptides, such as P11-4, create three-dimensional scaffolds that emulate enamel's protein matrix, promoting fluorapatite deposition. Randomized clinical trials indicate that P11-4 outperforms fluoride varnishes in remineralizing white spot lesions by integrating minerals into enamel prisms, offering a targeted approach to early caries reversal [16].

## PAMAM dendrimers:

Poly(amidoamine) (PAMAM) dendrimers, functioning as artificial proteins, enhance remineralization by sequestering calcium and phosphate ions, supporting intrafibrillar mineralization in both dentin and enamel. When functionalized with antimicrobial agents like honokiol, PAMAM dendrimers also inhibit cariogenic bacteria, addressing caries at its source [17]. These dual-action systems combine structural repair with bacterial control, making them promising for minimally invasive dentistry [18].

## Bioactive glass and hydrogels:

Bioactive glass (BAG) incorporated into hydrogels releases calcium, phosphate and fluoride ions, elevating oral pH to favor remineralization. Recent studies highlight BAG's ability to form hydroxy carbonate apatite, resembling natural enamel, with applications in treating dentin hypersensitivity and repairing early lesions. These gels dissolve within 30-50 minutes, delivering ions efficiently to demineralized areas and enhancing the enamel's resistance to acid challenges. Additionally, biomimetic enamel-like coatings, inspired by natural amelogenesis, are being explored to protect and repair enamel surfaces, showing promise in preclinical studies [19]. In summary, biomimetic strategies, including amelogenin-derived peptides, chitosan hydrogels, self-assembling peptides, PAMAM dendrimers and bioactive glasses, offer advanced solutions for enamel repair by emulating natural biomineralization. Continued research and clinical trials are crucial to overcome translational barriers and establish these methods in clinical practice, paving the way for next-generation caries prevention and regenerative dentistry [13]. Some of these biomimetic principles are being advanced even further through the use of nanotechnology, which enables precision delivery and control at the molecular level [20].

## Nanotechnology in remineralization:

The preservation and repair of early enamel lesions through remineralization remain central to preventive dentistry today. Recent progress in nanotechnology offers new avenues for delivering mineralizing agents with greater precision, thanks to their ability to mimic natural enamel at the nanoscale. This review outlines recent directions in the use of nanomaterials for enamel remineralization, focusing on nano-hydroxyapatite, calcium phosphate-based nanoparticles, advanced nanogels and silver nanoparticles [21].

## Nano-Hydroxyapatite (nHA):

nHA particles are engineered to resemble the natural crystalline framework of tooth enamel. Research across laboratory models and clinical studies indicates that nHA can integrate into weakened enamel zones, forming a protective layer and supplying essential ions that rebuild mineral density. Several reviews highlight that toothpastes containing nHA may be as effective as fluoride products for arresting early demineralization, with added safety for young patients due to their excellent biocompatibility [22].

## Calcium phosphate nanoparticles:

Beyond nHA, other nano-form calcium phosphates, such as amorphous calcium phosphate (ACP) and functionalized tricalcium phosphate (fTCP), have been blended into dental care formulations. These nanoparticles serve as localized ion reservoirs, helping to restore enamel by depositing new mineral phases. Evidence suggests that fTCP used with fluoride can boost remineralization results by controlling how ions interact. Additionally, bioactive glass nanoparticles have been developed to steadily release ions to support remineralization of early lesions [23].

## Smart nanogels and responsive systems:

Another promising approach uses "smart" nanocarriers that adjust to oral conditions. Chitosan-based nanogels that deliver ACP combined with antibacterial peptides have demonstrated the potential to limit bacterial activity while boosting mineral recovery during acid challenges [24]. Likewise, dental sealants enhanced with pH-sensitive nanoparticles can release calcium and phosphate ions specifically when acidity increases, providing protection only when the risk of demineralization is highest. This kind of responsive delivery minimizes unnecessary ion exposure and supports targeted enamel repair [25].

## Silver Nanoparticles (AgNPs):

AgNPs are of interest due to their potent antibacterial function. By disrupting the formation of bacterial biofilms and limiting acid production, they indirectly help protect enamel surfaces. Recent studies in children show that AgNP treatments can effectively arrest early carious lesions, often with less discoloration than traditional silver diamine fluoride applications [26]. Overall, nanotechnology adds new dimensions to how early caries can be managed. Materials such as nano-hydroxyapatite and calcium phosphate nanoparticles directly aid mineral replacement, while smart carriers and pH-responsive systems deliver ions precisely when needed [27]. Silver nanoparticles add a robust antibacterial effect, reducing caries risk at its source. Continued research and clinical evaluation are needed to confirm the long-term benefits and patient outcomes of these innovative techniques [28]. The precision and responsiveness of nanomaterials naturally lead to the development of "smart" dental systems that actively sense and respond to oral conditions [29].

## Smart materials and pH-modulating systems:

With the objective to preserve teeth and advance oral health, smart dental materials are made to react intelligently to physiological shifts and local environmental cues. In order to prevent cavities, encourage mineralization and safeguard tooth structures, advancements have recently been made in the development of smart dental materials with antibacterial and remineralizing properties that react to the local pH of the mouth. Dental smart materials are designed to react dynamically to the acidic environment that causes tooth demineralization. These stimuli-responsive devices provide targeted remineralization and decrease cariogenic biofilms by combining pH-triggered ion release with antibacterial qualities [30].

## PH-responsive calcium-phosphate composites:

Ca^2+^ and PO_4_^3-^ ions are preferentially released by amorphous calcium phosphate (NACP) nanoparticles implanted in resin composites when the local pH drops below the critical threshold (~5.5). *in vitro* studies show that substantial ion release at pH 4 and modest release at neutral pH protect the mineral reservoir. In contrast to commercial composites, smart NACP composites showed a dentin remineralisation rate of about 50% during the course of a typical demineralization/remineralization cycle of 4-8 weeks. Furthermore, a human in situ investigation revealed that the mineral loss in the enamel margins adjacent to smart composites was one-third that of the controls [30].

## Dual-Function NACP + Quaternary Ammonium Monomers:

Dimethylaminohexadecyl methacrylate (DMAHDM), a pH-activated antibacterial monomer, is added to resin to improve its performance. In acidic environments, DMAHDM exhibits potent antibacterial activity, breaking down Streptococcus mutans biofilms. Composite resins preserved enamel integrity by keeping the pH of the biofilm above 6 when paired with NACP and protein-repellent methacryloyloxyethyl phosphorylcholine (MPC), but ordinary adhesives caused the pH to decrease to about 4.2. In a study using 3 weight percent DMAHDM and 30% NACP, enamel hardness increased by two times when compared to controls under a continuous biofilm acid assault [25].

## Antibacterial pH-triggered Sealants:

Significant Ca and PO_4_ ions were liberated by resin sealants made with 20 wt% NACP and 5 wt% DMAHDM without sacrificing mechanical performance. Scanning Electron Microscopy with Energy Dispersive X-ray Spectroscopy (SEM-EDX) and polarised light microscopy investigations verified that these formulations decreased the loss of enamel surface hardness in pH cycling experiments. The combination of ion release and antibacterial activity significantly reduced enamel demineralisation surrounding sealants [31].

## Multifunctional composites with rechargeability:

To extend bioactivity, advanced composites combine rechargeable ion systems with agents including Poly(amidoamine) Dendrimers (PAMAM) and silver nanoparticles, as well as MPC, DMAHDM and NACP. Research shows that even after several recharge cycles, there is still antibacterial potency and persistent ion release. PAMAM and NACP composites, for example, demonstrated improved mineral development, dentin hardness and acid neutralisation compared to NACP alone [32].

## Biomimetic peptides:

pH-responsive self-assembling peptides, such as P11-4, serve as nucleation sites for the deposition of hydroxyapatite and create scaffolds in early carious lesions. These peptides reinforce enamel structures in a way similar to natural mineralisation by assembling and promoting mineral regeneration in response to acidic lesions [33]. In conclusion, acid- triggered antibacterial activity (via DMAHDM), pH-responsive ion release (mostly from NACP), protein-repellent components (MPC) and biomimetic scaffolds (*e.g.*, P11-4) are all combined in smart dental materials. Acidic buffering, biofilm suppression, targeted remineralisation and flexibility in response to changes in the oral environment are all provided by this multipurpose approach. The outcome was the use of minimally invasive techniques to restore mineral integrity and lower the risk of subsequent caries [34, 35]. Smart materials offer responsive and preventive benefits, but their clinical use depends on understanding current technologies, emerging advances and their integration into daily practice [36].

## Clinical applications and future perspectives:

Among the clinically available options for enamel remineralization, agents such as bioactive glass (BAG), nanohydroxyapatite (nHA) and casein phosphopeptide-amorphous calcium phosphate fluoride-based compounds (CPP-ACPF) have been widely used. In an *in vitro* study, the remineralization potential of these agents was assessed through scanning electron microscopy (SEM) and surface microhardness (SMH) testing. The results showed that nHA demonstrated the highest remineralization effectiveness, followed by BAG and CPP-ACPF [37]. In addition to these established approaches, there are emerging technologies with promising clinical potential. One example is PAMAM dendrimers, which mimic the natural action of amelogenin in guiding hydroxyapatite crystal organization. When functionalized with antimicrobial compounds such as honokiol, these biomimetic platforms offer not only enamel remineralization but also prevention of carious lesions. However, challenges related to large-scale synthesis and commercialization remain to be addressed [38]. Another innovative system involves hydrogels, which are three- dimensional networks of hydrophilic polymers capable of absorbing aqueous solutions and encapsulating bioactive agents. When enriched with calcium, phosphate and fluoride ions, these hydrogels can promote the formation of fluorapatite aggregates with enamel-like morphology. Materials such as gelatin, agarose and chitosan have been explored due to their structural and chemical similarity to the enamel matrix and the incorporation of antimicrobial agents such as silver or GL13K peptides further enhances their remineralizing and antibacterial properties [38]. Electrospun mats composed of amorphous calcium phosphate and polyvinylpyrrolidone (ACP/PVP) represent another innovative strategy. These mats act as reservoirs of calcium and phosphate ions, essential for remineralization. When exposed to fluoride-containing artificial saliva, the fibers become hydrated and form a mineralizing hydrogel that facilitates the crystallization of fluoridated hydroxyapatite, with morphology resembling that of natural enamel [39]. Although cariology science has progressed significantly, particularly in the understanding of the oral microbiome and non-invasive remineralization a strategy, translating this knowledge into evidence-based clinical protocols remains a major challenge. This is largely due to the high complexity and costs associated with conducting clinical trials and obtaining regulatory approvals. Furthermore, these technologies must be cost-effective, scalable and clinically applicable, requiring clear strategies for implementation, clinician training and professional adoption incentives [39].

## Conclusion:

Contemporary remineralization strategies are evolving from conventional fluoride therapies to innovative biomimetic, nanotechnology and smart material approaches. These methods not only aim to replace mineral loss but also replicate the structural and functional properties of enamel. Although promising, their clinical application is limited by cost, regulatory requirements and the need for long- term clinical validation. Continued translational research will be essential to bridge the gap between laboratory success and everyday dental practice.
